# Surgical results of Ahmed valve implantation combined with intravitreal triamcinolone acetonide injection for preventing choroidal detachment

**DOI:** 10.1186/1471-2415-15-13

**Published:** 2015-01-30

**Authors:** Hua Wang, Hong Chen, Yue Qi, Ning-li Wang

**Affiliations:** Beijing Tongren Eye Center, Beijing Tongren Hospital, Capital Medical University, Beijing Ophthalmology & Visual Science Key Lab, NO.1 Dongjiaominxiang street, Dongcheng District, Beijing, 100730 China; Beijing Institute of Ophthalmology, NO.17 Chongneihougou street, Beijing, 100005 China

**Keywords:** Glaucoma, Triamcinolone acetonide, Ahmed valve implantation

## Abstract

**Background:**

To investigate the surgical results of Ahmed valve implantation with intravitreal triamcinolone acetonide injection (IVTA) in patients with aphakic or pseudophakic glaucoma.

**Methods:**

Fifty-nine patients who underwent Ahmed valve implantation were consecutively recruited from November 2005 to August 2011 at the Beijing Tongren Hospital. In the IVTA group (29 eyes), 4 mg of TA was injected into the vitreous cavity during Ahmed valve implantation was performed. In the control group (30 eyes), only Ahmed valve implantation was performed. The primary outcome was intraocular pressure (IOP). The visual acuity (VA), supplemental medical therapy and complications were evaluated as secondary outcomes.

**Results:**

The IVTA and control groups were followed up for a mean of 22.7 and 31.4 months, respectively. IOP decreased in both groups at 1 day, 1 week, 1 month and the final follow-up points after the surgery. However, there was no significant difference between 2 groups at all intervals (P > 0.05). The rate of shallow anterior chamber and choroidal detachment in IVTA group was significantly lower than control group (6.9% vs. 40%, P = 0.007; 0% vs. 26.7%, P = 0.009).

**Conclusions:**

Ahmed valve implantation combined IVTA seems to be effective to reduce the incidence of complications for treatment of aphakic or pseudophakic glaucoma.

## Background

As the introduction of the Molteno implant [1], valve implantation has become one of the most effective therapies for all forms of glaucoma. However, simple valve implantation often causes severe complications, such as choroidal detachment, shallow anterior chamber, intraocular hemorrhage and retinal detachment subsequently [2, 3]. According to previous studies, the incidence of choroidal detachment after valve implantation could reach to 8 ~ 22% [4–6], which exerts severe damage to the eye and leads to surgery failure and visual loss.

Triamcinolone acetonide (TA), as a long-term glucocorticoid, has been proved to be effective for treating macular edema associated with diabetic retinopathy (DR) and ischemic central retinal vein occlusion (CRVO) [7, 8]. In addition to its effect on lowering inflammation and improving visual acuity in patients with DR and CRVO, intravitreal TA injection (IVTA) has been proposed as a potential adjuvant treatment for neovascular glaucoma (NVG) [9, 10]. We previously reported that IVTA was an effective and safe method to treat retinal detachment with choroidal detachment [11].

In this retrospective study, we compared the surgical results between Ahmed valve implantation with and without an IVTA in patients with aphakic or pseudophakic glaucoma.

## Methods

### Patients and inclusion criteria

This retrospective, comparative study was approved by the Institutional Review Board of Beijing Tongren Hospital Affiliated to Capital Medical University. Patients who underwent Ahmed valve implantation were consecutively recruited from November 2005 to August 2011at the BeijingTongren Hospital in Beijing, China. Patients included in the study should meet all the following criteria: 1) Patients aged 18 to 75 years with aphakic or pseudophakic glaucoma, who had previous glaucoma surgery and/or cataract surgery. Cataract surgeries must be transparent corneal incision, intact posterior capsular and no vitreous body into anterior chamber. 2) IOP was more than 22 mmHg even after the maximal usage of antiglaucoma medications. 3) All patients were in late stage of the disease, with the diagnostic criteria as follows: C/D > 0.8, tubular or island of visual field. Patients with other glaucoma diseases (glaucoma secondary to vitrectomy or penetrating keratoplasty. etc.) were excluded. The procedures were fully explained to each patient, and each provided written informed consent.

### Surgery technique

**Figure 1:**
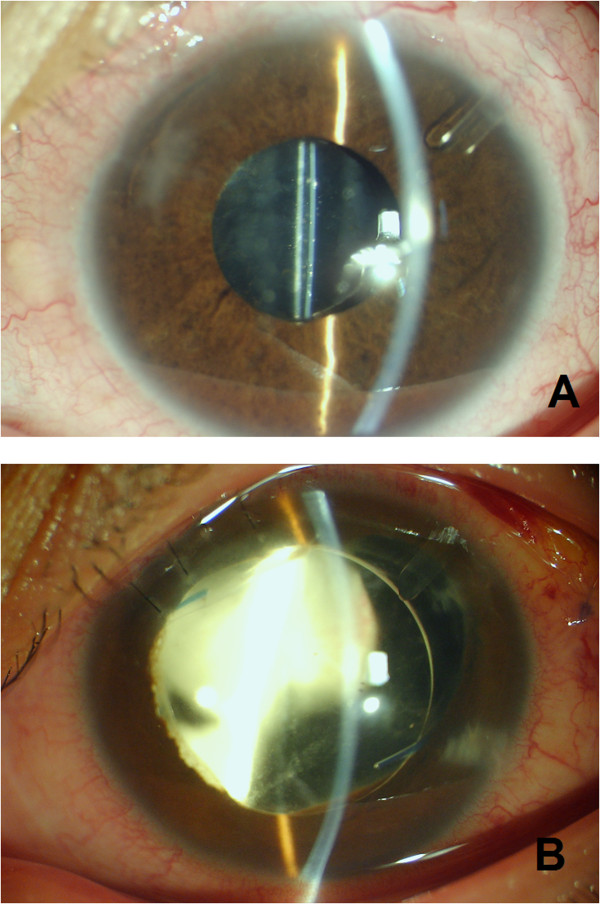
**Ahmed valve implantation combined with intravitreal triamcinolone acetonide injection seen by slitlamp biomicroscopy.** Ahmed valve implantation alone **(A)** and with triamcinolone acetonide injection **(B)**.

All procedures were performed by the same surgeon (Hong Chen) under sub-Tenon anaesthesia. A fornix-based conjunctival incision was made and Tenon capsule was dissected with a spring scissors. A 2.0 mm × 4.0 mm cellulose sponge pledget soaked with 0.04% (0.4 mg/ml) mitomycin C was pleased below the equatorial conjunctive for 5 min. The Ahmed Valve (New World Medical Inc, California, USA) was secured to the sclera in the superotemporal quadrant 8–10 mm back from the limbus. An entrance site into the anterior chamber was made under the scleral flap at the beginning of the blue scleral-limbal interface with a 23-G needle. The tube of the valve was then passed through this 23-G opening into the anterior chamber (Figure 1A). For TA group, TA (4 mg, 0.1 ml) was injected 3.5–4 mm away from the limbus (Figure 1B). The conjunctival and Tenon capsule incisions were closed at the limbus with a 10–0 nylon.

### Surgical outcomes

Visual acuity, IOP, number of antiglaucoma medications and complications were investigated, assessed, and recorded at each visit. Defining the time of operation as baseline, each patient was followed for 1 day, 1 week, 1 months and last visit.

### Statistical analysis

Visual acuity was expressed in logarithm of the minimum angle of resolution (logMAR) values. Standard values were used where Snellen values were unobtainable (no light perception [NLP], 3.0; hand movements, 2.0; light perception [LP], 2.3; and counting fingers, 1.7). All data analyses were completed with the SAS 9.1.3. (SAS Institute Inc, North Carolina, USA). Continuous variables were expressed as mean ± standard deviation (SD). Categorical variables were described with frequency (percentage). To compare differences between the study groups we used the student *t* test, the Mann-Whitney U test, the Wilcoxon signed-rank test, the chi-square test, and the Fisher exact test. *P*-values less than 0.05 were considered as statistically significant.

## Results

### Baseline characteristics

A total of 59 patients were included in this study, 29 eyes received Ahmed valve implantation with IVTA and 30 eyes received only Ahmed valve implantion without IVTA. In TA group, among a total of 29 patients, 18 patients were pseudophakic eyes and 11 patients were aphakic eyes. Of 30 patients in the control group, 16 patients were pseudophakic eyes and 14 patients were aphakic eyes. Baseline characteristics were presented in Table 1. No significant difference was found between the 2 groups in all categories.

**Table 1:** **Baseline characteristics of both study groups**

	IVTA group (n = 29)	Control group (n = 30)	P value
Age (y)	48.4 ± 18.96	46.2 ± 18.53	0.662
Sex (Male: Female)	17:12	14:16	0.358
Number of preoperative medications	3.69 ± 1.0	3.10 ± 1.32	0.059
Lens			0.497
Pseudophakic	18	16	
Aphakic	11	14	
Preoperative IOP (mm Hg)	31.6 ± 13.13	36.4 ± 12.65	0.113
Follow-up (mo)	22.7 ± 14.6	31.4 ± 19.9	0.060

IVTA = intravitreous triamcinolone acetonide injection.

### Intraocular pressure control and medical therapy

**Figure 2:**
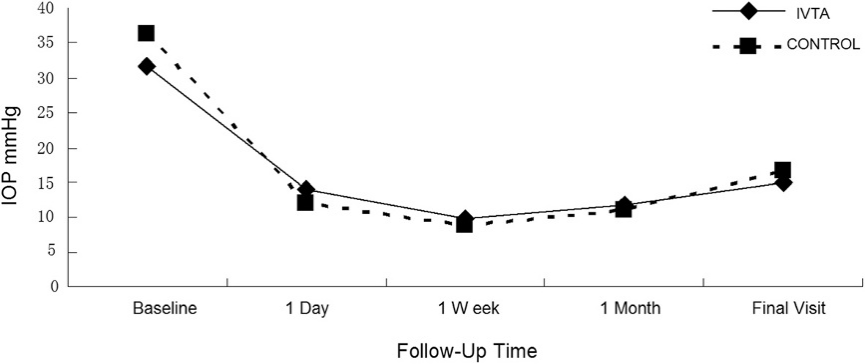
**Mean intraocular pressure (IOP) after Ahmed valve implantation in eyes with or without intravitreal triamcinolone acetonide injection.**

The preoperative mean of IOP was 31.6 ± 13.13 mm Hg in the IVTA gourp and 31.4 ± 19.9 mm Hg in the control group (P = 0.113 by *t* test). Compared with preoperative IOP, the two groups showed a statistically significant IOP decrease at all postoperative time (P < 0.05 by Wilcoxon signed-rank test). However, there was no significant difference between the two groups at each time point (P > 0.05 by Mann-Whitney U test, Table 2 and Figure 2). Compared with the preoperative number of medications, both groups showed a statistically significant decrease in the number of medications at last visit (P < 0.001). However, the mean number of medications of the 2 group did not show statistically significant differences with each other (0.45 ± 0.91 vs. 0.43 ± 0.82; P = 0.945).

**Table 2:** **Intraocular pressure changes during the study period**

Time	IVTA group (n = 29)		Control group (n = 30)		P value
	Mean ± SD	Range	Mean ± SD	Range	
Baseline	31.6 ± 13.13	15–59	36.4 ± 12.65	14–60	0.113
Day 1	14.0 ± 7.84	7–45	12.1 ± 8.97	5–50	0.076
Weak 1	9.83 ± 3.21	7–20	8.94 ± 2.37	5–14	0.555
Month 1	11.9 ± 2.96	7–20	11.1 ± 3.33	5–22	0.238
Last follow up	14.9 ± 4.98	8 ~ 25	16.7 ± 7.70	7–27	0.399

### Visual acuity

Visual acuity results are shown in Table 3. At final visit, there was no significant difference in logMAR snellen visual acuity (P = 0.63 by paired *t* test) between the 2 groups. Visual acuity was improved from baseline in 17 (58.6%) patients in the IVTA group and 11 (36.7%) in the control group. Moreover, visual acuity was decreased in 3 (10.4%) patients in the IVTA group and 10 (33.3%) patients in control group. There was no significant difference between the two groups (P = 0.08 by chi-square test).

**Table 3:** **Visual acuity results**

	IVTA group (n = 29)	Control group (n = 30)	P value
**LogMAR (mean ± SD)**			
Baseline	1.30 ± 0.65	1.21 ± 0.76	0.63
Last visit	1.05 ± 0.79	1.24 ± 0.90	0.39
Changed	0.33 ± 0.30	0.30 ± 0.34	0.68
Number of changed			0.08
Improved	17 (58.6%)	11 (36.7%)	
Unchanged	9 (31.0%)	9 (30%)	
Decreased	3 (10.4%)	10 (33.3)	

### Complications

Table 4 details the complications in each group, and no statistically significant difference was found complications in vitreous hemorrhage, hyphema, valve obstruction and uncontrollable IOP. However, more cases of shallow anterior chamber was found in the control group than IVTA group (6.9% vs. 40%, P = 0.007). Moreover, choroidal detachment was noted in 8 (26.7%) cases of the control group and no case in IVTA group (P = 0.009).

**Table 4:** **Postoperative complications**

	IVTA group (n = 29)	Control group (n = 30)	P value
Shallow anterior chamber	2 (6.9%)	12 (40.0%)	0.007
Choroidal detachment	0 (0.0%)	8 (26.7%)	0.009
Vitreous hemorrhage	1 (3.4%)	0 (0.0%)	0.986
Hyphema	1 (3.4%)	2 (6.7%)	1.000
Valve obstruction	1 (3.4%)	3 (10%)	0.629
Uncontrollable IOP	4 (13.8%)	4 (13.3%)	1.000

In addition, 1 case of mild vitreous hemorrhage and 1 case of anterior chamber hemorrhage were occurred in the TA group and 2 cases of anterior chamber hemorrhage were noted in the control group. All of these 4 patients were relieved after conservative treatment. Each groups had 4 patients that still had much higher IOP than the normal value. One of them was performed ophthalmectomy owing to the uncontrollable IOP in control group and others were control after treating with anti-glaucoma medicines.

## Discussion

Refractory glaucoma, including neovascular glaucoma (NVG), uveitis or traumatic secondary glaucoma, aphakic or pseudophakic glaucoma, iridocorneal endothelial (ICE) syndrome, and failed trabeculectomy are all potentially blinding diseases in which it is often difficult to control intraocular pressure (IOP), even with maximum doses of antiglaucoma medication [12]. Ahmed valve implantation, with or without suture, is thought to be an effective method to treat patients with refractory glaucoma [6, 9, 13]. However, some postoperative complications such as shallow anterior chamber and choroidal effusion, always bother the patients and clinicians. Thus, in this study, we pointed out that the Ahmed valve implantation combined with IVTA maybe a good choice for preventing these complications.

Even with Ahmed glaucoma drainage valve implants, shallow anterior chambers often occur, and following complications such as choroidal detachment, suprachoroidal hemorrhage, peripheral iris synechiae, or cataract has been reported [14, 15]. Park et al. [15] found that shallow anterior chambers and choroidal detachment followed by hypotony were observed more with younger age, myopia, and less previous intraocular surgery. Moreover, many research demonstrated that there exist various pathological factors accounting for these complications such as postoperative inflammation, vessel dilatation in choroid membrane and plasma leakage. Vitreous hemorrhage, retinal detachment and blindness always occur in serious complications cases, which lead to the failure of surgery and visual loss. Although a series of precaution measures were performed to prevent these complications, for example, viscoelastic injection in anterior chamber, drainage tube ligation, autogenous or heterogenous sclera flap transplantation, none of these measures is effective sufficiently.

Penfold et al. [16] firstly reported the application of TA for treating age-related macular degeneration (AMD) in 1995. Since then, a number of studies affirmed the therapeutic effect of IVTA for treating kinds of hyperplastic, hydropigenous and angiogenic eye diseases. Some studies reported that IVTA was a useful remedy for suppressing uveitis and improve the success rate of retinal detachment with choroidal detachment [17, 18]. The mechanisms of TA acted in valve implanation maybe that: 1) Reducing early postoperative dilatation of blood vessels, maintaining the vascular permeability and preventing choroidal vascular leakage as well as choroidal detachment. 2) Inhibiting the proliferation of fibroblast and differentiation of pigment epithelial cell, thus reducing the formation of hyperplastic fibrosis which will block the valve’s drainage. 3) Controlling the inflammation process and preventing tube’s obstruction.

With respect to postoperative complications, compared with the control group, combination with TA injection could significantly reduce the severity and prevalence of flat anterior chamber and choroidal detachment. In our study, the incidence rate of flat anterior chamber and choroidal detachments was 40% and 26.7% respectively in control group, which were similar to previous studies [4–6]. 1 of 8 choroidal detachments in control group turned blindness and 2 of them had to receive the vitrectomy. All of the three patients had visual loss and other 3 patients with choroidal detachments had to receive TA injection for treating choroidal detachments. By contrast, IVTA group had 6.9% of flat anterior chamber and none choroidal detachment occurred, which was less frequent than that in control group. Furthermore, there were 2 patients with mild intraocular hemorrhage in combination group, resulting from inexperienced surgical operation, who turned better with conservative treatment finally.

Based on previous study, one notable side-effect of IVTA for treating ocular fundus diseases was that it caused increased IOP and the prevalence was about 28.8 ~ 40.4% [18–23]. However, in our study, IOP did not increase in TA group, no matter at early or late stage after the surgery. The reasons could be attributed as follows. Firstly, a much higher dosage of TA (more than 4 mg) was given in previous study [16]. In our study, the TA dose we used was 4 mg, which was a much safer dosage. Secondly, TA alone was injected into the hermetic vitreous in previous studies; but in the current study, we applied TA combining with valve drainage that part of it could outflow via the valve, reducing its concentration and action time in the vitreous. Finally, the decreased IOP caused by valve implantation surgery might counteract the effect of increased IOP caused by TA injection.

Our study is limited by the retrospective nature of the study and also by short follow-up time. Moreover, the sample size of this study only allowed us to detect fairly large differences between the groups. Therefore, a large series study with randomized controlled design is needed to provide us more accurate outcome predictions.

## Conclusions

In conclusion, we believed that Ahmed valve implantation combined with IVTA could significantly reduce the prevalence and severity of postoperative flat anterior chamber and choroidal detachment for pseudophakic or aphakic patients with glaucoma.
